# Post-treatment with H12-(ADP)-liposomes after LPS challenge ameliorated coagulopathy and critical organ injury in rats

**DOI:** 10.1186/s40635-026-00906-4

**Published:** 2026-05-08

**Authors:** Kohsuke Hagisawa, Osamu Ishida, Hiroyuki Nakashima, Shinji Takeoka, Yuji Morimoto, Manabu Kinoshita

**Affiliations:** 1 Department of Physiology, National Defense Medical College, 3-2 Namiki, Tokorozawa, Saitama 359-8513 Japan; 2 Department of Surgery, National Defense Medical College, Tokorozawa, Japan; 3 Department of Immunology and Microbiology, National Defense Medical College, Tokorozawa, Japan; 4 Department of Life Science and Medical Bioscience, Graduate School of Advanced Science and Engineering, Waseda University, Tokyo, Japan

**Keywords:** Platelet–leukocyte aggregates, Consumptive coagulopathy, And Critical organ injuries

## Abstract

**Background:**

Liposomes coated with fibrinogen γ-chain (HHLGGAKQAGDV, H12) peptide and encapsulating adenosine-diphosphate (ADP) [H12-(ADP)-liposomes] can augment platelet aggregation via glycoprotein IIb/IIIa receptors displayed on activated platelets. H12-(ADP)-liposomes release ADP, which is metabolized into adenosine that has tissue-protective effects. This study evaluated the life-saving efficacy of post-treatment with H12-(ADP)-liposomes in rats with LPS-induced coagulopathy and critical organ injuries.

**Methods:**

LPS (10 mg/kg) was administered intraperitoneally to rats. The rats were then treated with an intravenous injection of either H12-(ADP)-liposomes or normal saline (vehicle control) 4 h later.

**Results:**

Post-treatment with H12-(ADP)-liposomes significantly shortened the coagulation time compared to the vehicle treatment at 8 h after LPS challenge and reduced expression of CD62P, a marker of platelet activation, on CD61^+^ platelets at 12 h. H12-(ADP)-liposome post-treatment also normalized the elevated levels of neutrophil elastase (complex) at 6–24 h and citrullinated histone H3 in bronchoalveolar lavage fluid at 24 h. H12-(ADP)-liposome-treated rats showed reductions in the pathological injury score for the lungs and kidneys at 8 h. The survival rates of rats given H12-(ADP)-liposomes were markedly improved 24 h after LPS challenge relative to vehicle-treated rats (50% vs. 21%, *p* = 0.038).

**Conclusions:**

These findings suggest that post-treatment with H12-(ADP)-liposomes ameliorates LPS-induced coagulopathy and neutrophil activation, thereby improving critical organ injuries and survival in LPS-challenged rats.

**Supplementary Information:**

The online version contains supplementary material available at 10.1186/s40635-026-00906-4.

## Introduction

The poor survival rate of septic patients with consumptive coagulopathy and critical organ injuries remains a significant problem in critical care settings [1]. Septic shock often causes coagulopathy involving excessively activated platelets [2]. The consumption and diminished supply of platelets and coagulation proteins typically results in oozing from wounds, but profuse hemorrhaging can also occur [1]. Comprehensive, specific, and effective treatments for consumptive coagulopathy have yet to be established.

In terms of the pathophysiology of consumptive coagulopathy with septic shock, activated platelets synergistically interact with neutrophils to form neutrophil extracellular traps (NETs) at injured endothelium. In NETs, neutrophils release molecules termed damage-associated molecular patterns (DAMPs) that stimulate formation of platelet–leukocyte aggregates (PLA). These chain reactions exacerbate host conditions in critically ill patients. Circulating cell-free DNA and histones released from neutrophils during sepsis also induce PLA and activate coagulation cascades [3, 4] that are both correlated with coagulopathy severity and mortality [3, 5]. Platelets orchestrate the inflammatory response by regulating the adhesion of leukocytes during the inflammatory process, and consequently platelets are consumed by PLA [4, 6]. Therefore, we focused on activated platelets in PLA as a therapeutic target for consumptive coagulopathy.

We developed liposome-based artificial platelets coated with synthetic HHLGGAKQAGDV (H12) peptides that correspond to the C terminus of the fibrinogen γ-chain and encapsulate the physiological platelet agonist adenosine 5'-diphosphate (ADP) [7]. We previously reported that H12-(ADP)-liposomes may be an effective platelet substitute for thrombocytopenic rabbits [8, 9]. The H12 peptide includes the primary recognition site for the GPIIb/IIIa receptor displayed on the surface of platelets and targets sites of vascular injury where platelets are activated. Upon binding to platelet aggregates, H12-(ADP)-liposomes release the encapsulated ADP, which can be hydrolyzed by nucleotidases expressed by endothelial cells and leukocytes, and that circulates in the plasma to maintain nucleotide homeostasis [10]. We also found that adenosine produced by ADP metabolism could protect mice from fatal blast lung injury with hemorrhage [11]. The released ADP stimulates platelet aggregation via P2Y signaling and may be metabolized into adenosine, which protects against organ injury via purinergic signaling [10, 12].

The one-shot lipopolysaccharide (LPS) challenge model faithfully recapitulates septic shock. LPS drives septic shock pathogenesis induced by Gram-negative bacterial infections, and the challenge can induce symptoms similar to those experienced by patients or animals with septic shock [13, 14]. Although many studies have used this simple injection model to evaluate possible therapies and/or drugs, most involved pretreatment or delivery of the treatment immediately after LPS challenge [15–18]. However, in clinical settings, intensive care for septic patients usually begins at the worst time point for the clinical symptoms. LPS challenge models typically exhibit catastrophic systemic symptoms/responses at more than 3 h after the challenge [19]. We thus investigated effective post-treatments for sepsis/septic shock in rats starting the intervention at 4 h after LPS challenge. Here, we examined the effects of post-treatment with H12-(ADP)-liposomes on reducing consumptive coagulopathy and critical organ injuries induced by LPS challenge in rats.

## Methods

### Animals and reagents

All experiments were performed in accordance with the National Defense Medical College institutional ethical guidelines for experiments involving animals. Protocols were approved by the Committee for Animal Research at the National Defense Medical College under ethical approval number #22033. Male Sprague Dawley (SD) rats, 14–15 weeks old (450 ± 23 g; Japan SLC, Hamamatsu, Japan), were housed at 22–24 °C with a 12-h light/dark cycle and free access to food and water. Our reporting on research involving animals follows ARRIVE guidelines.

### Preparation of H12-(ADP)-liposomes

H12-(ADP)-liposomes were prepared as a diluted suspension with phosphate buffered saline (PBS) following the same procedure as previously reported [8, 9, 12]. The total lipid concentration was 20 mg/mL. The average diameter of the H12-(ADP)-liposomes was 216 ± 58 nm, and the concentration of encapsulated ADP was 0.112 mg/mL.

### H12-(ADP)-liposome treatment for LPS-challenged rats

**Fig. 1:**
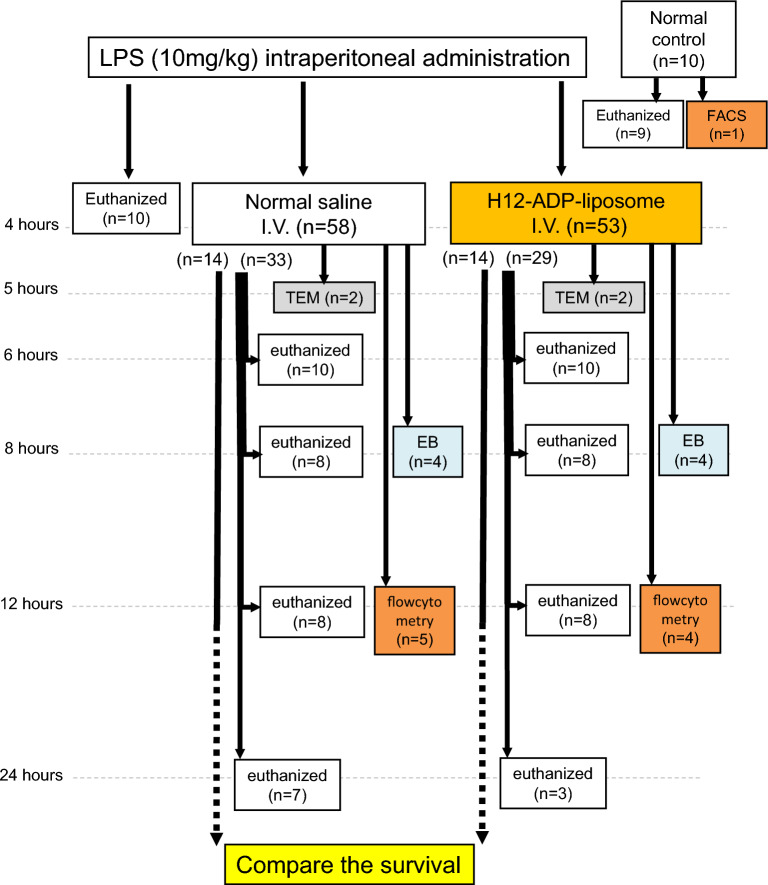
Experimental protocol. All rats received an intraperitoneal administration of LPS (10 mg/kg) followed by either a vehicle or H12-(ADP)-liposomes via the tail vein 4 h later. A total of 28 rats were evaluated for prognosis. Another 72 rats were euthanized at the indicated time points (4, 6, 8, 12, and 24 h) following the LPS challenge to collect blood samples and organs for pathological analysis. Additionally, 21 more rats were used for TEM, FACS, or Evans Blue examinations. Animals euthanized at the 24-h time point were prepared to compensate for animals that died during the prognosis study

LPS (L3129-25MG, Sigma-Aldrich, St. Louis, MO) was administered intraperitoneally to rats at a dose of 10 mg/kg (Fig. 1). At 4 h after the LPS challenge, the rats received either H12-(ADP)-liposomes (LPS + H12-(ADP)-liposomes; 40 mg/1 mL/kg, *n* = 14) or normal saline as the vehicle (LPS + Vehicle; 1 mL/kg, *n* = 14) via the tail vein to monitor/compare the survivals. Ten rats were administered LPS and then euthanized 4 h after the LPS challenge to collect blood samples and organs for pathological analysis. Another 62 rats were administered LPS, followed by H12-(ADP)-liposomes (*n* = 33) or normal saline (*n* = 29). These rats were euthanized at 6, 8, 12, and 24 h after the LPS challenge to collect blood samples and organs for pathological analysis. The blood sampling volume for the complete blood count (CBC), biochemistry, coagulation, and ELISA analysis was approximately 6 mL. Concerning that this blood withdrawal could affect secondary blood cell activation and endothelial damage, which interfere with the transmission electron microscopy study, flow cytometer analysis, and Evans Blue study, additional 21 rats were administered LPS followed by H12-(ADP)-liposomes (*n* = 11) or normal saline (*n* = 10).

### Hematological analysis and coagulation activity of whole blood

Coagulation activity was evaluated using a Sonoclot coagulation analyzer (model SCP2; Sienco, Morrison, CO). This system determines clotting time (CT) that corresponds to the time needed for fibrin formation to begin [20].

Platelet function was assessed by multiple electrode impedance platelet aggregometry using a Multiplate® Analyzer (Roche Diagnostics, Mannheim, Germany) whereas platelet aggregation was assessed by a collagen test [21].

Blood samples were collected in tubes containing 10% sodium citrate (vol/vol) and centrifuged at 500×*g* at 4 °C for 10 min. These samples were used for measurement of plasma fibrinogen levels and plasma antithrombin (AT) activity that was carried out at the Sanritsu Zelcova Laboratory (Tokyo, Japan).

Commercially available rat FDP/D-dimer (LS-F55531; LSBio, Seattle, WA), rat thrombin antithrombin (LS-F40596; LSBio, Seattle, WA), and rat syndecan-1 (NBP2-76611; Novus Biologicals, CO) ELISA kits were used according to the manufacturer’s instructions.

### Histopathological examinations

At 6 and 8 h after LPS challenge (2 and 4 h after H12-(ADP)-liposome treatment or normal saline treatment), rats were euthanized using isoflurane. The lungs and kidneys were collected and fixed in 4% paraformaldehyde for 24 h before embedding in paraffin. Sections (4 µm-thick) were cut from the embedded tissues and stained with hematoxylin–eosin (HE). Pathological injury scores for acute lung injury (ALI) were measured as described by Kulkarni et al. [22]. In brief, the following features were evaluated: (A) Neutrophils counted in the alveolar airspaces (1–5 counts, 1 point, > 5 counts, 2 points); (B) Alveolar walls and interstitium (1–5 counts, 1 point, > 5 counts, 2 points); (C) Hyaline membranes (1 count 1 point, > 1 count, 2 points), (D) Proteinaceous debris filling the airspaces (1 count 1 point, > 1 count 2 points), and (E) Septal thickening (2–fourfold greater than the control: 1 point, > fourfold greater than the control, 2 points). Pathological injury scores for ALI were then determined using the equation: [(20 × A) + (14 × B) + (7 × C) + (7 × D) + (2 × E)]/(number of fields × 100). Pathological injury scores for acute kidney injury (AKI) were measured as described by Gupta et al. [23]. In brief, severity scale values were assigned a score ranging from 0 to 3 (0 = normal, 1 = mild, 2 = moderate, and 3 = severe pathology) for each of 3 variables: tubular vacuolization, tubular dilatation, and tubular cast formation.

An additional eight rats (four in the LPS + vehicle group and four in the LPS + H12-(ADP)-liposome group) were prepared for the Evans Blue evaluation 8 h after LPS challenge. Briefly, a 0.5% solution of Evans Blue (EB) was injected intravenously and allowed to circulate for 30–60 min. To ensure that only extravasated dye was measured, the pulmonary circulation was perfused thoroughly with PBS through the right ventricle until the lungs appeared white, indicating the removal of intravascular blood. The harvested lung tissue was weighed, incubated at 60 °C for 24 h with 500 μL of formamide for every 100 mg of tissue to fully extract the dye, and then weighed again. The EB concentration was determined using a spectrophotometer with a peak wavelength of 620 nm. Background correction at 740 nm was used as a reference to correct for tissue turbidity [24].

### Transmission electron microscopy examinations

To prevent the H12-(ADP)-liposomes from collapsing due to excessive blood cell aggregation over time, the rats treated with H12-(ADP)-liposomes after the LPS challenge were euthanized with isoflurane and the lung specimens were isolated as soon as possible. However, the glutaraldehyde perfusion to fix and excise the lung tissues took 1 h. This process was completed 5 h after the LPS challenge. The specimens were examined by transmission electron microscopy, as previously described [25, 26].

### Examination of bronchoalveolar lavage fluid (BALF)

Rats treated with H12-(ADP)-liposomes (*n* = 6), or normal saline (*n* = 4) were euthanized with isoflurane 6 or 8 h after LPS challenge and BALF was obtained. In brief, the chest cavity was dissected to expose the lung and the trachea. An 18-gauge catheter (Surflo SR-OX1832CA Terumo, Tokyo, Japan) was gently inserted into the trachea and 30 mL/kg of chilled saline was administered and aspirated slowly through the catheter. The total albumin concentration of the BALF was determined using the Bradford method (Quick Start™ protein assay kit, Bio-Rad, Hercules, CA). A commercially available rat HNE/Neutrophil Elastase CLIA kit (LS-F31099; LSBio, Seattle, WA) was used to measure neutrophil elastase complex levels, and a rat citrullinated histone H3 ELISA kit (MBS7255106; MyBioSource, San Diego, CA) was used to measure citrullinated histone H3 levels in the BALF.

### Flowcytometric analyses

Platelet activation and PLA formation were assessed using flow cytometry and monoclonal antibodies that included phycoerythrin (PE)-labeled anti-mouse/rat CD62P (P-selectin) antibody (clone; RMP-1, BioLegend, San Diego, CA) for platelet P-selectin, allophycocyanin (APC) anti-mouse/rat CD61 (clone; 2C9.G2 (HMβ3-1), BioLegend, San Diego, CA) for platelets, and fluorescein isothiocyanate (FITC) mouse anti-rat CD45 (clone; OX-1, BD Biosciences, Franklin Lakes, NJ) for leukocytes [27, 28]. Whole blood samples were collected in tubes with 3.2% citrate and diluted tenfold with PBS, followed by the addition of a defined amount of the indicated fluorescent antibody and incubation at room temperature for 20 min. PE anti-mouse/rat CD62P was used with APC anti-mouse/rat CD61 to analyze CD62P expression on platelets, whereas FITC mouse anti-rat CD45 was used with APC anti-mouse/rat CD61 to assess PLA formation. The samples were subsequently fixed with ThromboFix platelet stabilizer (Beckman Coulter, Brea, CA) and stored at 4 ℃ in a light-shielded environment until measurement.

A NovoCyte Flow Cytometer System (Agilent Technologies, Inc., Santa Clara, CA) was used for flow cytometric analysis. Blood samples were fixed with formaldehyde and stained with fluorescence-labeled antibodies. The final blood dilution for analysis was 1:100. Because platelets are smaller than red blood cells, the pore size used for analysis was set as small as possible to avoid overlapping with other blood cells. Therefore, the flow rate was 14 µL/min, which is substantially slower than that typically used for immune cell analysis. Based on the forward and side scatter histogram, platelets were identified as in an area ‘A’, (Supplemental Fig. 1) and 100,000 events corresponding to platelets were acquired.

Platelet CD62P expression was analyzed for CD61-positive cells and used to calculate the number of CD62P-positive cells (Supplemental Fig. 2). PLA analysis was performed by calculating the population of CD61-positive cells among CD45-positive cells. Leukocytes were identified by CD45-positive cells in the cell cluster, which has more FSC-SSC signals than those for erythrocytes (Supplemental Fig. 3). Among the CD45-positive cells, a cell population positive for the platelet marker CD61 appeared in LPS-treated rats (Supplemental Fig. 4). This distinct population was considered to be PLA, as reported previously [29]. The appropriate isotype control antibodies were used for each sample to avoid detection of nonspecific binding, and One-comp compensation beads (Thermofisher Scientific, Waltham, MA) were used for fluorescent spill-over correction.

### Statistical analysis

Statistical analyses were performed using the ystat2000 software package (Igakutosho Shuppan, Tokyo, Japan). To compare animal prognoses, we determined sample sizes using a power analysis with G*Power software based on our preliminary data. With an effect size of 1.14, alpha = 0.05, and power = 0.8, the required sample size was 14 rats per group. Sample sizes for comparison of hematological parameters were also determined by power analysis using G*Power software based on our preliminary data. With an effect size of 1.4, alpha = 0.05, and power = 0.8, the required sample size was 10 rats per group. Survival rates were compared with a log-rank test. Statistical evaluations between two groups were compared using a Student's *t* test, and any other statistical evaluations were compared using one-way analysis of variance followed by a Bonferroni post hoc test. *p* < 0.05 was considered to be statistically significant.

## Results

### LPS-induced consumptive coagulopathy

**Fig. 2:**
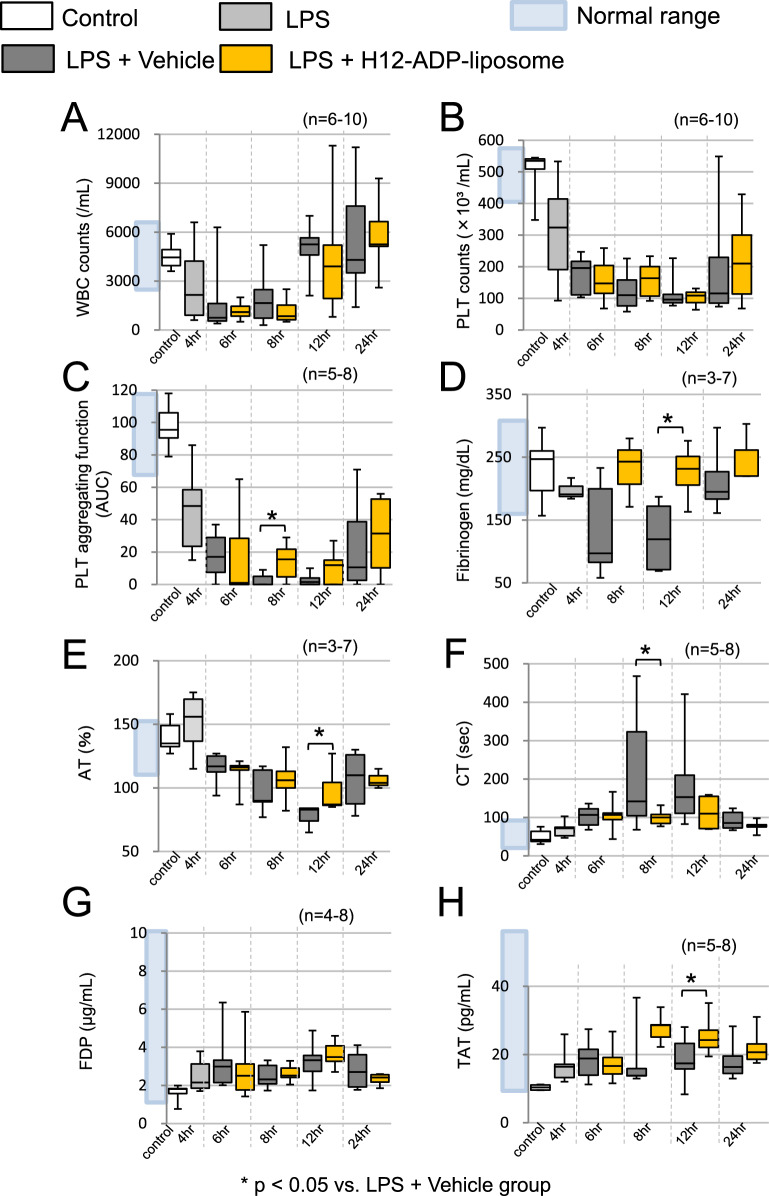
Effect of H12-(ADP)-liposomes on blood cell counts, platelet function and coagulation factors in LPS-challenged rats. **A**, **B** Leukocytopenia and thrombocytopenia were induced by LPS challenge as evidenced by changes in WBC (**A**) and platelet (**B**) counts in blood samples taken from rats euthanized at the indicated time point. **C** Platelet aggregating function was preserved in part in the LPS + H12-(ADP)-liposome group. **D** Hypofibrinogenemia was induced by LPS challenge and the LPS + H12-(ADP)-liposome group had suppressed fibrinogen consumption. **E** H12-(ADP)-liposome treatment suppressed AT consumption. **F** H12-(ADP)-liposome treatment ameliorated prolonged CT. **G** Amounts of fibrinogen degradation product (FDP) were elevated after LPS challenge in both vehicle- and liposome-treated groups. **H** Levels of thrombin-antithrombin (TAT) complexes were elevated after LPS challenge. Open bars correspond to the untreated control group. The light gray bars correspond to the group observed 4 h after the LPS challenge. Dark gray bars correspond to the group treated with LPS + H12-(ADP)-liposomes 4 h after the LPS challenge. Yellow bars correspond to the group treated with LPS + H12-(ADP)-liposomes 12 h after the LPS challenge. Asterisks indicate significant differences (*p* < 0.05)

Leukocytopenia and thrombocytopenia were detected 6 h after LPS challenge and continued until 12 h after LPS challenge (Fig. 2A, B). Platelet aggregating activity, as assessed with a Multiplate analyzer, was diminished at 8–12 h after the LPS challenge as compared to the untreated control (4 ± 4 vs. 98 ± 13 AUC; *p* < 0.01, *n* = 5–8; Fig. 2C). Plasma fibrinogen levels decreased significantly and were around one-half the level of that for the untreated control group 12 h after the LPS challenge (123 ± 62 vs. 231 ± 49 mg/dL; *p* = 0.01, *n* = 4, 7). Antithrombin (AT) consumption was also detected (78 ± 8 vs. 140 ± 12%; *p* < 0.01, *n* = 7; Fig. 2D, E). The clotting time (CT) was significantly prolonged 8 h after the LPS challenge compared to the untreated control (219 ± 156 vs. 50 ± 17 s; *p* = 0.007, *n* = 7, 8; Fig. 2F). LPS challenge was associated with a significantly elevated plasma thrombin-antithrombin complex (TAT) concentration 12 h after LPS challenge compared to the untreated control (19 ± 7 vs. 10 ± 1 pg/mL; *p* = 0.04, *n* = 5, 7; Fig. 2H), which substantiates that the LPS-challenged rats developed hyper-coagulative, but not hyper-fibrinolytic, coagulopathy.

### Post-treatment with H12-(ADP)-liposomes ameliorated platelet function and coagulation activity in LPS-challenged rats

H12-(ADP)-liposome post-treatment did not influence platelet counts (Fig. 2B), but platelet aggregating activity was maintained to some extent 8–12 h after LPS challenge in the LPS + H12-(ADP)-liposome group relative to the LPS + Vehicle group 8 h after LPS challenge (14.2 ± 11.6 vs. 3.8 ± 3.8 AUC; *p* = 0.04, *n* = 5, 6; Fig. 2C). Meanwhile, prolongation of CT was significantly suppressed in the LPS + H12-(ADP)-liposome group relative to the LPS + Vehicle group at 8 h (100 ± 20 vs. 219 ± 156 s, *p* = 0.04, *n* = 6, 7) after LPS challenge (Fig. 2F). Plasma FDP and TAT concentrations increased similarly in both groups after the LPS challenge (Fig. 2G and H), but the LPS + H12-(ADP)-liposome group showed ameliorated hypofibrinogenemia (226 ± 48 vs. 124 ± 62 mg/dL; *p* = 0.02, *n* = 4) and suppressed AT consumption (97 ± 18 vs. 78 ± 8%; *p* = 0.01, *n* = 6. 7) when compared with the LPS + Vehicle group 12 h after the LPS challenge (Fig. 2D and E).

### Post-treatment with H12-(ADP)-liposomes mitigated ALI

**Fig. 3:**
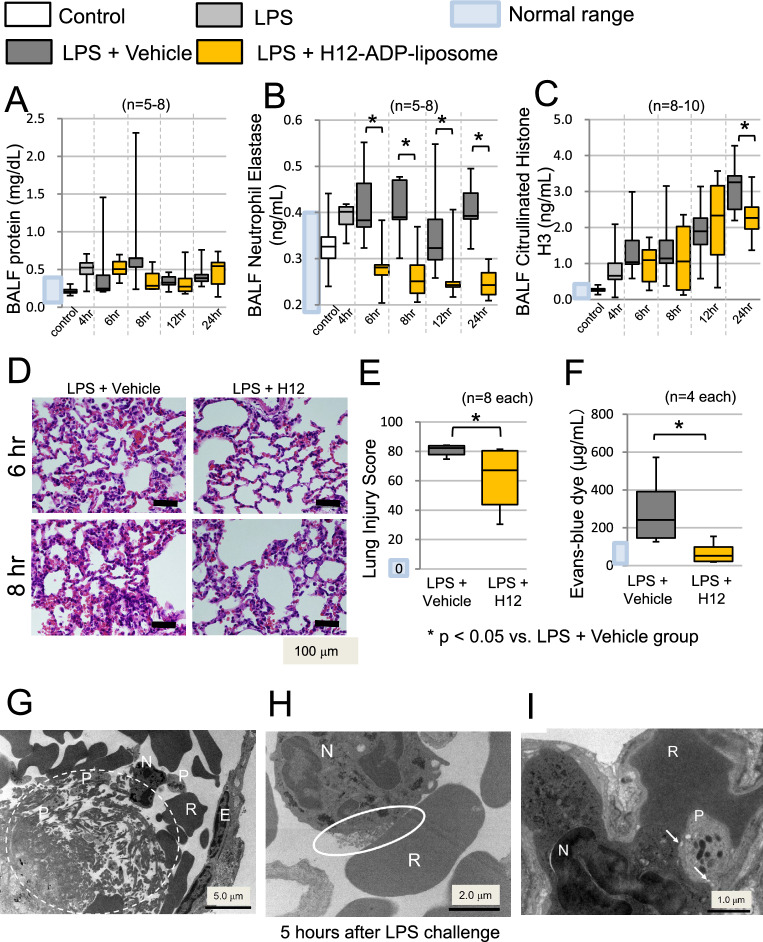
Effect of H12-(ADP)-liposomes on lungs in LPS-challenged rats. **A** H12-(ADP)-liposomes did not suppress exudative protein levels in BALF, but they did reduce, **B** BALF neutrophil elastase activity and **C** levels of BALF citrullinated histone H3. **D** Alveolar neutrophil infiltration observed in the lungs of animals in the LPS + vehicle group was suppressed in animals that received H12-(ADP)-liposomes. **E** The pathological injury score was significantly higher in the LPS + Vehicle group than in the LPS + H12-(ADP)-liposome group, which also had reduced, **F** Evans blue dye extravasation relative to the LPS + vehicle group 8 h after the LPS challenge. Transmission electron microscopy showed. (**G**) Platelet aggregation with PLA and fibrin deposition (white dashed circle) that occurred due to disseminated intravascular coagulation. **H** Neutrophil degranulation (white oval) was observed. **I** Arrows indicate H12-(ADP)-liposomes accumulating on PLA in lung capillaries. P: platelet; N: neutrophil; E: endothelium; R: erythrocyte. Scale bars in panels **A**–**C** represent 5.0 µm, 2.0 µm, and 1.0 µm, respectively. Open bars correspond to the untreated control group. The light gray bars correspond to the group that was observed 4 h after the LPS challenge. Dark gray bars correspond to the LPS + vehicle group. Yellow bars correspond to the LPS + H12-(ADP)-liposome group. Asterisks indicate significant differences (*p* < 0.05)

To investigate the tissue-protective effect of H12-(ADP)-liposomes, bronchoalveolar lavage fluid (BALF) was taken from the animals at multiple time points after LPS challenge. The LPS + H12-(ADP)-liposome group did not have reduced amounts of exudating protein in the BALF (Fig. 3A), but did have significantly reduced neutrophil elastase (complex) levels at 6 h (0.28 ± 0.05 vs. 0.42 ± 0.08 ng/mL; *p* = 0.004, *n* = 7), 8 h (0.27 ± 0.06 vs. 0.40 ± 0.07 ng/mL; *p* = 0.01, *n* = 5), 12 h (0.26 ± 0.06 vs. 0.36 ± 0.10 ng/mL; *p* = 0.04, *n* = 6, 7), and 24 h (0.25 ± 0.04 vs. 0.41 ± 0.05 ng/mL; *p* = 0.001, *n* = 7, 8) after LPS challenge (Fig. 3B). The elevation of citrullinated H3 levels in BALF was significantly reduced by H12-(ADP)-liposome treatment 24 h after LPS challenge (2.3 ± 0.6 vs. 3.1 ± 0.7 ng/mL; *p* < 0.05, *n* = 8) (Fig. 3C).

Pathological examinations of lung tissues excised 6 and 8 h after LPS challenge revealed massive neutrophil infiltration into the alveolar and interstitial spaces of the lungs in the LPS + Vehicle group. In contrast, the LPS + H12-(ADP)-liposome group exhibited modest neutrophil infiltration 6 and 8 h after LPS challenge (Fig. 3D). The pathological lung injury score [30] of the LPS + H12-(ADP)-liposome group was also significantly lower than that for the LPS + Vehicle group 8 h after LPS challenge (62 ± 21 vs. 81 ± 4; *p* < 0.05, *n* = 8; Fig. 3E). Consistent with this finding, lungs from animals in the LPS + H12-(ADP)-liposome group had reduced Evans blue dye extravasation relative to the LPS + Vehicle group 8 h after LPS challenge (70 ± 64 vs. 296 ± 206 μg/mL; *p* < 0.05, *n* = 4; Fig. 3F).

### Ultrastructural analysis of lung tissue in LPS-challenged rats

Transmission electron microscopy of lung tissue taken from rats 4 h after LPS challenge showed fibrin deposits with platelet aggregation and platelet–neutrophil complexes attached to capillary endothelial cells (Fig. 3G). In pulmonary capillaries, extracellular granules from a neutrophil were found (Fig. 3H, white circle). Meanwhile, in pulmonary capillaries taken from rats in the LPS + H12-(ADP)-liposome group 5 h after LPS challenge, liposomes were detected in PLA (Fig. 3I, arrows).

### Post-treatment with H12-(ADP)-liposomes ameliorated AKI

**Fig. 4:**
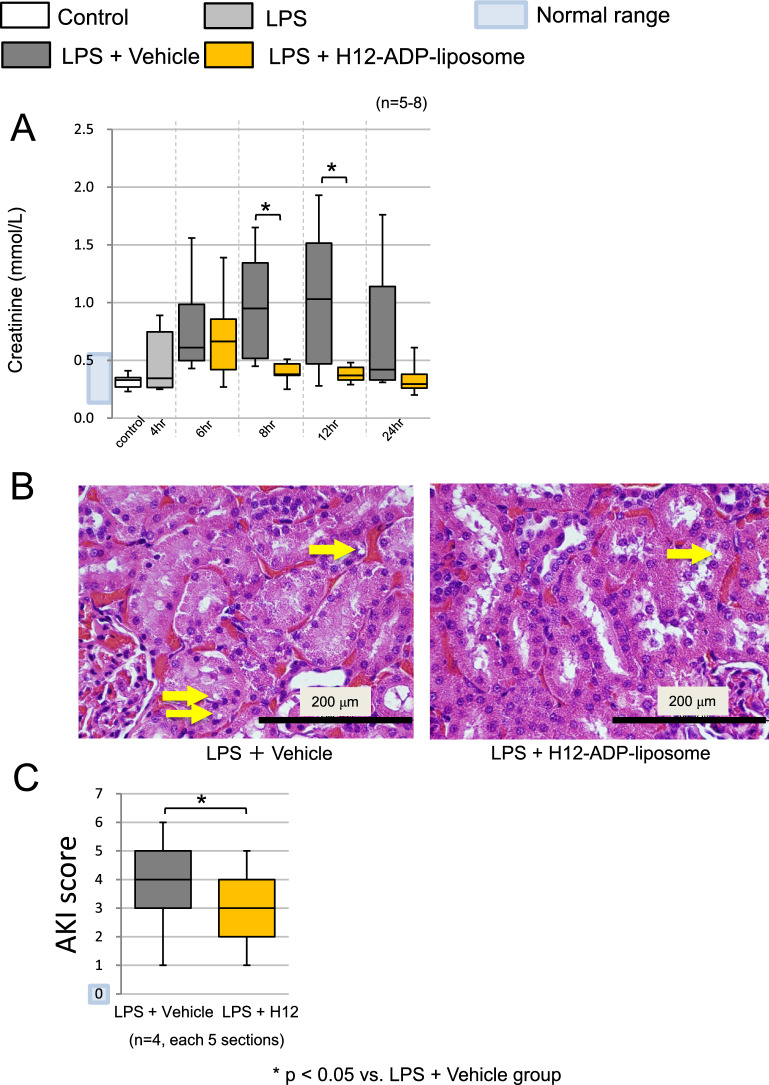
Effect of H12-(ADP)-liposomes on the kidneys of LPS-challenged rats. **A** H12-(ADP)-liposome treatment suppressed increases in serum creatinine levels following LPS challenge. **B** Vacuolization of distal tubular cells, a characteristic of AKI, was reduced in the LPS + H12-(ADP)-liposome group (yellow arrows). **C** The AKI score of the LPS + Vehicle group was significantly higher than that of the LPS + H12-(ADP)-liposome group. Open bars correspond to the untreated control group. The light gray bars correspond to the group that was observed 4 h after the LPS challenge. Dark gray bars correspond to the LPS + vehicle group. Yellow bars correspond to the LPS + H12-(ADP)-liposome group. Asterisks indicate significant differences (*p* < 0.05)

Rats in the LPS + Vehicle group developed severe AKI, whereas those in the LPS + H12-(ADP)-liposome group had suppressed elevations in serum creatinine 8 h (0.4 ± 0.1 vs. 1.0 ± 0.5 mmol/L; *p* = 0.01, *n* = 6, 7) and 12 h (0.4 ± 0.1 vs. 1.0 ± 0.7 mmol/L; *p* = 0.03, *n* = 5, 6) after LPS challenge (Fig. 4A). Pathological examinations of the kidneys at 8 h after LPS challenge showed vacuolization (arrows) of distal tubular cells that are characteristic of AKI. In contrast, the LPS + H12-(ADP)-liposome group had only modest vacuolization (Fig. 4B). The pathological score for tubular injury [23] showed a significant reduction in the LPS + H12-(ADP)-liposome group compared to LPS + Vehicle group (2.9 ± 1.0 vs. 4.2 ± 1.5; *p* = 0.03, *n* = 4 animals with 5 sections each) 8 h after LPS challenge (Fig. 4C).

### Post-treatment with H12-(ADP)-liposomes ameliorated septic shock and increased survival rates

**Fig. 5:**
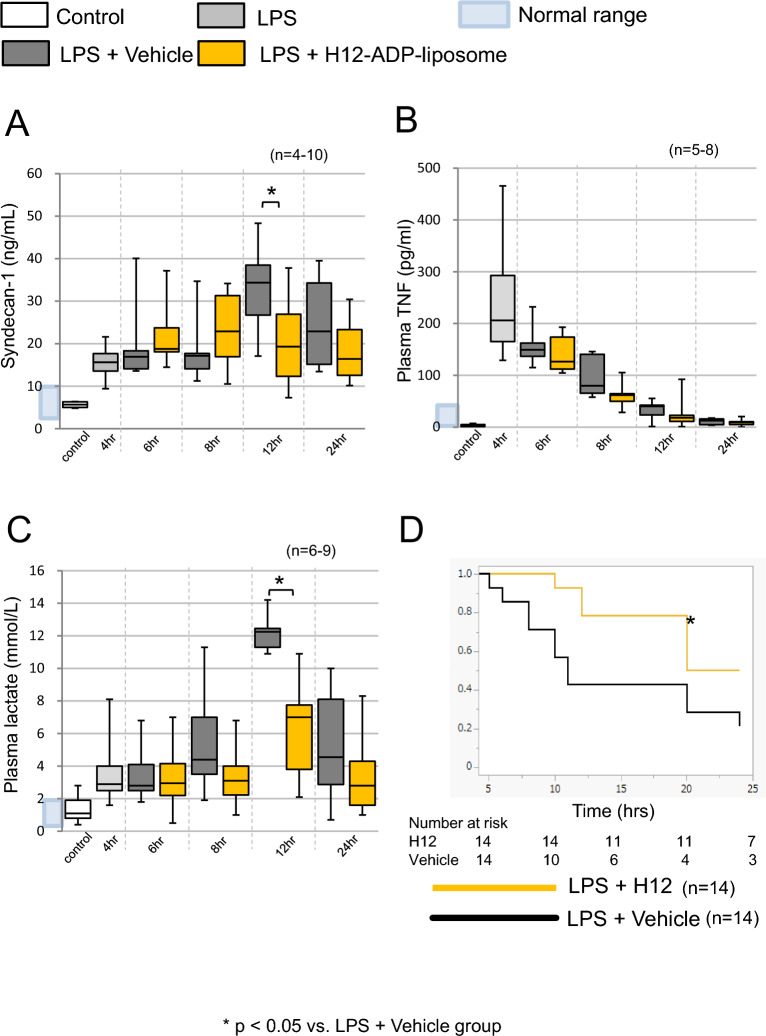
H12-(ADP)-liposome treatment mitigates parameters associated with septic shock and increases survival of LPS-challenged rats. **A** Syndecan-1 levels were increased by LPS challenge, and this increase was suppressed in animals treated with H12-(ADP)-liposomes at 12 h after LPS challenge. **B** H12-(ADP)-liposome treatment partially suppressed elevations in plasma TNF levels. **C** H12-(ADP)-liposome treatment significantly suppressed increases in plasma lactate levels reflecting hypoxic stress. **D** H12-(ADP)-liposome treatment increased survival of rats exposed to LPS, Open bars correspond to the untreated control group. The light gray bars correspond to the group that was observed 4 h after the LPS challenge. Dark gray bars correspond to the group treated with LPS + H12-(ADP)-liposomes. Yellow bars correspond to the group treated with LPS + H12-(ADP)-liposome 4 h after LPS challenge. Asterisks indicate significant differences (*p* < 0.05)

Suppression of elevated plasma syndecan-1 levels were associated with less damage to the endothelium following H12-(ADP)-liposome treatment (Fig. 5A). The liver function data showed significant variation, such that no meaningful results were obtained (Supplemental Fig. 5C). Although H12-(ADP)-liposome post-treatment did not augment the elevation of plasma TNF induced by LPS challenge (Fig. 5B), it did suppress elevations in plasma lactate levels seen at 12 h after LPS challenge (6.2 ± 3.1 vs. 12.2 ± 1.2 mmol/L; *p* = 0.0005, *n* = 6, 7; Fig. 5C) and improved the acute survivals of rats after LPS challenge (50% vs. 21% survival at 24 h; *p* = 0.038, *n* = 14; Fig. 5D).

### Flowcytometric analyses revealed that H12-(ADP)-liposomes suppressed platelet activation and PLA formation

**Fig. 6:**
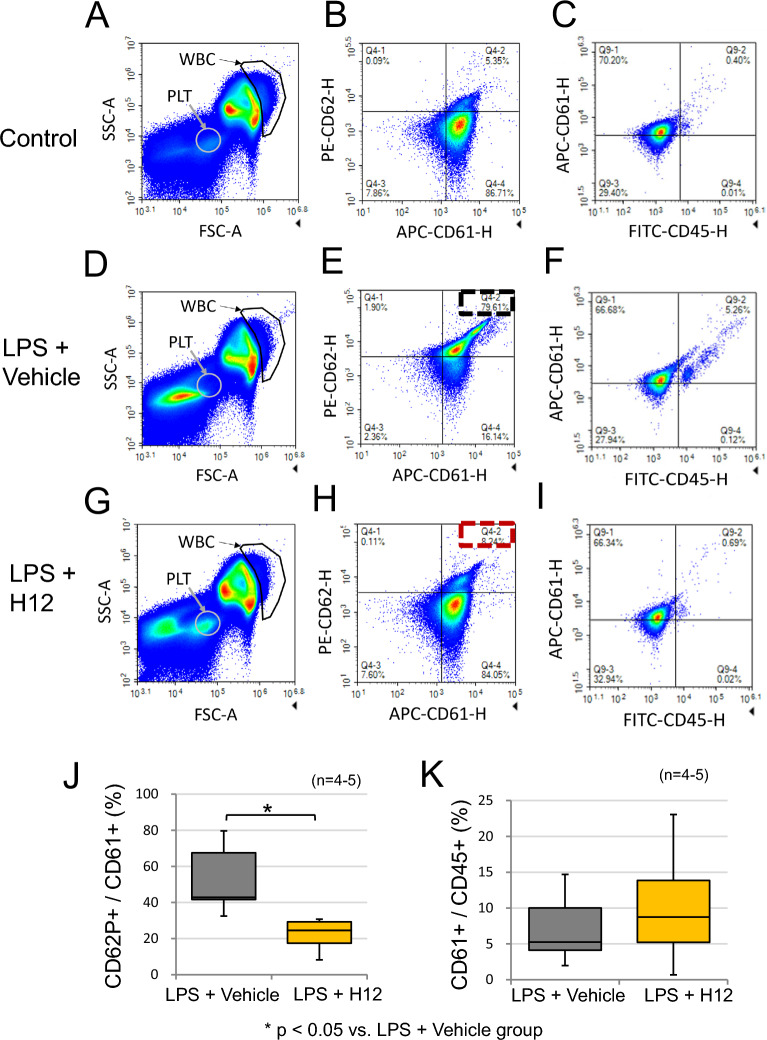
Flowcytometry analysis of platelet activation and PLA formation induced by LPS challenge. Whole peripheral blood samples were taken from rats in the different experimental groups 12 h after LPS challenge and analyzed. **A**, **D**, **G** The platelet fraction was selected based on cell size and complexity using forward and side scatter analysis (FSC-A and SSC-A, respectively, Supplementary Fig. 1). **B**, **E**, **H**, **J** Platelet fractions were developed into CD61-CD62P histograms (Supplementary Fig. 2). LPS increased the population of CD62P^+^ cells in the platelet fraction (black dashed square). H12-(ADP)-liposome treatment attenuated LPS-induced increases in CD62P^+^ in the platelet fraction (red square, panel **J**). **C**, **F**, **I**, **K** The WBC fraction was selected based on FSC-A and SSC-A and developed into CD45-CD61 histograms (Supplementary Figs. 3 and 4). LPS tended to increase the number of CD61^+^ and CD45^+^ double positive cells in the WBC fraction, suggesting formation of PLA. However, H12-(ADP)-liposome treatment did not significantly affect PLA formation (panel **K**). Asterisks indicate significant differences (*p* < 0.05)

To evaluate platelet activation, flow cytometric analyses were performed using whole blood samples. At 12 h after the LPS challenge in the LPS + Vehicle group, the activated CD62P^+^ fraction was predominant in the whole CD61^+^ platelet population, as evidenced by a 79.6% Q4-2 fraction (Fig. 6E, upper right, dashed box). Meanwhile, in the LPS + H12-(ADP)-liposome group, the CD62P^+^ population was reduced (8.24% Q4-2; Fig. 6H). Simultaneously, a CD61^+^ population among CD45 cells appeared, which was associated with PLA formation at 12 h after LPS challenge (Fig. 6F). The percentage of this population decreased with H12-(ADP)-liposome treatment (Fig. 6I). Indeed, statistical analysis revealed that the proportion of CD62P^+^ activated platelets decreased by nearly half in the H12-(ADP)-liposome group (22 ± 10%; *p* < 0.05, *n* = 4, 5; Fig. 6J) compared to the untreated LPS group (53 ± 19%). Although the percentage of CD61^+^ CD45^+^ PLA increased to 7% ± 5% after LPS challenge, this percentage did not differ significantly from that seen for the LPS + H12-(ADP)-liposome group (*p* = 0.49, *n* = 3, 4; Fig. 6K).

## Discussion

### Possible mechanisms of efficacy in post-treatment with H12-(ADP)-liposomes in LPS-challenged rats

To our knowledge, no other treatment has been able to effectively rescue animals from LPS-induced shock at time points when damage is most severe (i.e., at 4 h after LPS challenge). The findings of this study to examine the efficacy of H12-(ADP)-liposomes to protect animals following LPS challenge have potential clinical value. H12-(ADP)-liposomes were originally developed as platelet substitutes, but they also have certain tissue protective effects associated with the encapsulated adenosine. H12 attached on the liposome surface mimics the active site of fibrinogen and binds to activated platelets via GP IIb/IIIa receptors. In septic hosts, high amounts of platelet–leukocyte aggregation (PLA) products are present, and these aggregated platelets are presumably activated, and thus able bind to the administered H12-(ADP)-liposomes. The ADP encapsulated in H12-(ADP)-liposomes can also be quickly metabolized to adenosine that has protective anti-inflammatory effects on tissues. The results of the current study suggest that H12-(ADP)-liposomes could bind to activated platelets that are components of PLA, and that ADP-derived adenosine could suppress activation of neutrophils in PLA to result in down-regulation of neutrophil-involved inflammation in septic hosts. PLA, which is a target of H12-(ADP)-liposomes, is generated because of septic inflammation, thereby post-treatment with H12-(ADP)-liposomes can be effective in the presence of PLA, even after sepsis has developed.

### Levels of citrullinated histone H3 and neutrophil elastase in BALF decreased with post-treatment H12-(ADP)-liposomes in LPS-challenged rats

Transmission electron microscopy revealed that H12-(ADP)-liposomes were detected in PLA in pulmonary capillaries. Neutrophil elastase levels in BALF were suppressed in the H12-(ADP)-liposome group. Post-treatment with H12-(ADP)-liposomes in LPS-challenged rats also reduced levels of citrullinated histone H3 in BALF. Citrullinated histone H3 has been established as a biomarker for NET formation and a predictor of prognosis in septic patients [31, 32], while inhibition of NETs in sepsis by DNase was shown by McDonald et al. to reduce intravascular coagulation, microvascular circulation disturbance, and organ damage [33]. The suppression of citrullinated histone H3 and neutrophil elastase levels by H12-(ADP)-liposomes thus may contribute to the suppression of consumptive coagulopathy. Under this condition, platelets are primarily activated in alveolar capillaries to form PLAs and NETs.

### H12-(ADP)-liposomes suppressed excessive platelet activation and endotheliopathy

Flow cytometry results revealed that LPS-challenged rats given post-treatment H12-(ADP)-liposomes exhibited significantly reduced CD62P expression on activated platelets. Post-treatment with H12-(ADP)-liposomes effectively suppressed elevations in levels of plasma syndecan-1, which is recognized as a biomarker for endotheliopathy [34]. CD62P, known as P-selectin, is a specific adhesion molecule for the P-selectin glycoprotein ligand-1 (PSGL-1) on neutrophils. In severely injured hosts, multiple chemokines regulate neutrophil activation and recruitment, and platelets are recruited to inflammation sites, resulting in thromboinflammation and/or immunothrombosis including PLA [35, 36]. After this recruitment, PLAs bind to the endothelium by interacting with PSGL-1 on platelets and P-selectin on injured endothelium [37]. Granai et al. recently reported that these platelet–leukocyte interactions depend on the P-selectin-PSGL-1 axis, and that P-selectin hyperexpression indicates endothelial and platelet activation. This activation leads to micro- and macrothrombosis in the pathology of SARS-CoV-2 infection [38]. Therefore, CD62P is recognized as a potential therapeutic target for PLA-related (NET-related) diseases [39].

### H12-(ADP)-liposomes ameliorated consumptive coagulopathy

Plasma fibrinogen levels reflect the severity of sepsis/septic shock patients and predict outcomes [40]. CT prolongation is known to be associated with an increased risk of developing consumptive coagulopathy and increased mortality associated with such conditions [41]. Although H12-(ADP)-liposomes were injected into rats 4 h after LPS challenge, they nonetheless significantly improved LPS-induced hypofibrinogenemia, which supports the potential clinical application of these liposomes. H12-(ADP)-liposome post-treatment also alleviated CT prolongation, resulting in a favorable prognosis. These results suggest that H12-(ADP)-liposomes could play a crucial role in suppressing LPS-induced consumptive coagulopathy, even in a post-challenge setting. In addition to improving whole blood coagulation (e.g., CT), the H12-(ADP)-liposome treatment maintained coagulation factor levels. The estimated AT activity for animals in the LPS + H12-(ADP)-liposome group was 71.4% based on the ratio with the control value and is consistent with a study by Gando et al. who reported that patients showing AT activity levels ≥ 60% had better outcomes [42]. Meanwhile, Zipperle et al. reported that platelet-mediated hemostatic function is impaired with increasing PLA levels [43]. Taken together, H12-ADP-liposomes could ameliorate consumptive coagulopathy to achieve a favorable prognosis.

### H12-(ADP)-liposomes mitigated critical organ injuries

Morphological studies revealed that LPS challenge in rats resulted in intravascular coagulation in capillaries in the lungs at sites where PLA was associated with platelets. In addition, ALI and AKI occurred within 8 h of LPS challenge. Nevertheless, these pathological features were attenuated by H12-(ADP)-liposome post-treatment (at 4 h after LPS challenge) and were likely associated with both hemostatic and anti-neutrophil inflammatory effects. Functional studies of organs showed that H12-(ADP)-liposome treatment protected vascular permeability in lung capillaries, and renal function. Moreover, H12-(ADP)-liposome post-treatment suppressed levels of plasma lactate that reflect systemic oxygenized status. Overall, H12-(ADP)-liposome post-treatment improved acute survival in rats after LPS challenge.

## Limitation

This LPS model induces endotoxemia and simplifies secondary hematological and hemostatic reactions. However, it lacks two key features of human sepsis: bacterial dissemination and immune adaptation. Thus, further research is needed before H12-(ADP)-liposomes can be used to treat septic shock in humans.

There is a discrepancy between the number of enrolled animals and the number of blood samples. This discrepancy is associated with the difficulty in obtaining sufficient amounts of blood samples for ELISA due to hypovolemia induced by LPS administration.

The Syndecan-1 levels fall in the LPS + vehicle group after 24 h compared to 12 h without any treatment might be due to survival bias. In brief, the animals with severely elevated Syndecan-1 had more endothelial damage and were dead, so blood samples were unavailable.

In this study, direct attachment of H12-(ADP)-liposomes to PLA was only detected by ultrastructural examination. Future studies using confocal microscopy with fluorescent staining dyes will help elucidate the detailed functional relationship between PLA and H12-(ADP)-liposomes.

There were no findings of side effects of H12-(ADP)-liposomes such as thrombosis in this cohort, along with our previous studies. However, these liposomes may exacerbate ongoing thrombus formation in cases that have unstable coronary atherothrombotic plaques with highly activated platelets.

We used H12-(ADP)-liposomes for single bolus administration in our previous studies, because this agent was developed for prehospital treatment. If a single dose is insufficient, repeated dosing may be administered every 12 h based on the liposome half-life. As part of normal sepsis treatment, no volume was administered intravenously after LPS stimulation, except for H12-(ADP)-liposomes or the vehicle. However, the administration of a crystalloid infusion as part of standard sepsis treatment could significantly impact coagulation and endothelial dysfunction.

## Conclusion

In this study, H12-(ADP)-liposomes diminished the consumptive coagulopathy induced by LPS challenge and ameliorated critical organ injuries in rats. In this pathology, H12-(ADP)-liposomes were found in PLA, where they suppressed CD62P expression and maintained alveolar neutrophil elastase and plasma syndecan-1 within normal ranges. These results suggest that H12-(ADP)-liposomes may inhibit excessive platelet activation via ADP/adenosine signaling, thereby improving neutrophil function and vascular endothelial damage and relieving LPS-induced consumptive coagulopathy.

## Supplementary Information

Supplementary material 1. Supplemental Figure 1. Identification of platelets in a forward and side scatter histogram. Whole blood samples were stained with CD61 and treated with ThromboFix platelet stabilizer. Flow cytometric analysis was performed using a Novocyte flow cytometer. A histogram with cellular size (forward scatter by area, FSC-A) and complexity (side scatter by area, SSC-A) is displayed in the left panel. The X and Y axes are scaled logarithmically to clearly display smaller signals than erythrocytes, which are abundant (yellow gate in the left panel). Two clusters of smaller cells were identified as gate "A" and "B" (red and brown gates in the left panel). Gate A is positive for platelet marker CD61 compared with the isotype control antibody (right upper panel). Considering the cell size, which is smaller than erythrocytes, and CD61 expression, gate A is consistent with platelets. Supplemental Figure 2. Activated platelets identified with CD61 and CD62P. Platelets were gated in FSC-A and SCC-A, then developed into CD61 and CD62P histograms. After LPS treatment, the percentage of the CD61 and CD62P double-positive population increased compared to the untreated normal group (compare pink squares in the right upper and lower panels). Upregulation of the platelet activation marker CD62P indicates that LPS treatment induces platelet activation in peripheral blood. CD62P expression was compared with samples stained with isotype control antibodies (the upper and lower middle panels). Supplemental Figure 3. Identification of leukocytes in whole blood samples with CD45 and FSC/SSC histograms. The cellular size (FSC) and complexity (SSC) of leukocytes, including lymphocytes and polymorphonuclear cells, are higher than those of erythrocytes. In the FSC-SCC histogram (upper left panel), a cell cluster with a higher FSC-A signal (red gate; upper panels) than that of erythrocytes (yellow gate; lower panels) was identified. Pan-immune cell marker CD45 staining revealed that a small percentage of CD45-positive cells was detectable in the red gate (gray arrow in right upper panel), whereas no significant signal was detected in the yellow gate (right lower panels). These CD45-positive signals are consistent with leukocytes in the whole blood sample, and the cells remaining in the red gate, which are negative for CD45, are considered to be doublets or triplets of erythrocytes. CD45 expression levels were compared with samples stained with isotype control antibodies (the upper and lower middle panels). Supplemental Figure 4. Platelet–leukocyte aggregates identified with CD45 and CD61 histograms. The high FSC-A cell clusters (red gates in the upper and lower left panels), which include leukocytes, were developed into CD61-CD45 histograms (right panels). CD61-positive signals were detected in both the untreated normal and LPS-treated samples (gray arrows in the upper and lower panels), which suggests the presence of platelet–erythrocyte aggregates. However, CD61-CD45 double-positive cells were detected (red circle and black arrow in the right lower panel) only in the LPS-treated sample. Considering the cell size, which is larger than single erythrocytes, and the presence of both leukocyte and platelet markers, these are consistent with platelet–leukocyte aggregates (PLAs). CD61 expression was compared with samples stained with isotype control antibodies (the upper and lower middle panels). Supplemental Figure 5. Liver function. Rats in the LPS + H12-(ADP)-liposome group tended to have suppression of the elevation in AST and ALT. Open bars correspond to the untreated control group. Gray bars correspond to the group challenged with LPS 4 h after treatment. Black bars correspond to the LPS + vehicle group. Yellow bars correspond to the LPS + H12-(ADP)-liposome group.

## Data Availability

The data sets used and/or analyzed during the current study are available from the corresponding author on reasonable request.

## Abbreviations

ADP: Adenosine-diphosphate
AKI: Acute kidney injury
AKI: Acute lung injury
APC: Allophycocyanin
AT: Antithrombin (AT)
BALF: Bronchoalveolar lavage fluid
CT: Clotting time
DAMPs: Damage-associated molecular patterns
FITC: Fluorescein isothiocyanate
LPS: Lipopolysaccharide
NETs: Neutrophil extracellular traps
PLA: Platelet–leukocyte aggregates
TAT: Thrombin–antithrombin complex
