# Supplementary material for: Post-treatment with H12-(ADP)-liposomes after LPS challenge ameliorated coagulopathy and critical organ injury in rats
Source: Intensive Care Med Exp. 2026 May 8;14:61. doi: 10.1186/s40635-026-00906-4 (PMC13156337; doi:10.1186/s40635-026-00906-4)
Supplement: Supplementary file 1 — Supplementary material 1. Supplemental Figure 1. Identification of platelets in a forward and side scatter histogram. Whole blood samples were stained with CD61 and treated with ThromboFix platelet stabilizer. Flow cytometric analysis was performed using a Novocyte flow cytometer. A histogram with cellular size (forward scatter by area, FSC-A) and complexity (side scatter by area, SSC-A) is displayed in the left panel. The X and Y axes are scaled logarithmically to clearly display smaller signals than erythrocytes, which are abundant (yellow gate in the left panel). Two clusters of smaller cells were identified as gate "A" and "B" (red and brown gates in the left panel). Gate A is positive for platelet marker CD61 compared with the isotype control antibody (right upper panel). Considering the cell size, which is smaller than erythrocytes, and CD61 expression, gate A is consistent with platelets. Supplemental Figure 2. Activated platelets identified with CD61 and CD62P. Platelets were gated in FSC-A and SCC-A, then developed into CD61 and CD62P histograms. After LPS treatment, the percentage of the CD61 and CD62P double-positive population increased compared to the untreated normal group (compare pink squares in the right upper and lower panels). Upregulation of the platelet activation marker CD62P indicates that LPS treatment induces platelet activation in peripheral blood. CD62P expression was compared with samples stained with isotype control antibodies (the upper and lower middle panels). Supplemental Figure 3. Identification of leukocytes in whole blood samples with CD45 and FSC/SSC histograms. The cellular size (FSC) and complexity (SSC) of leukocytes, including lymphocytes and polymorphonuclear cells, are higher than those of erythrocytes. In the FSC-SCC histogram (upper left panel), a cell cluster with a higher FSC-A signal (red gate; upper panels) than that of erythrocytes (yellow gate; lower panels) was identified. Pan-immune cell marker CD45 staining revea [file 40635_2026_906_MOESM1_ESM.ppt]

## Slide 1
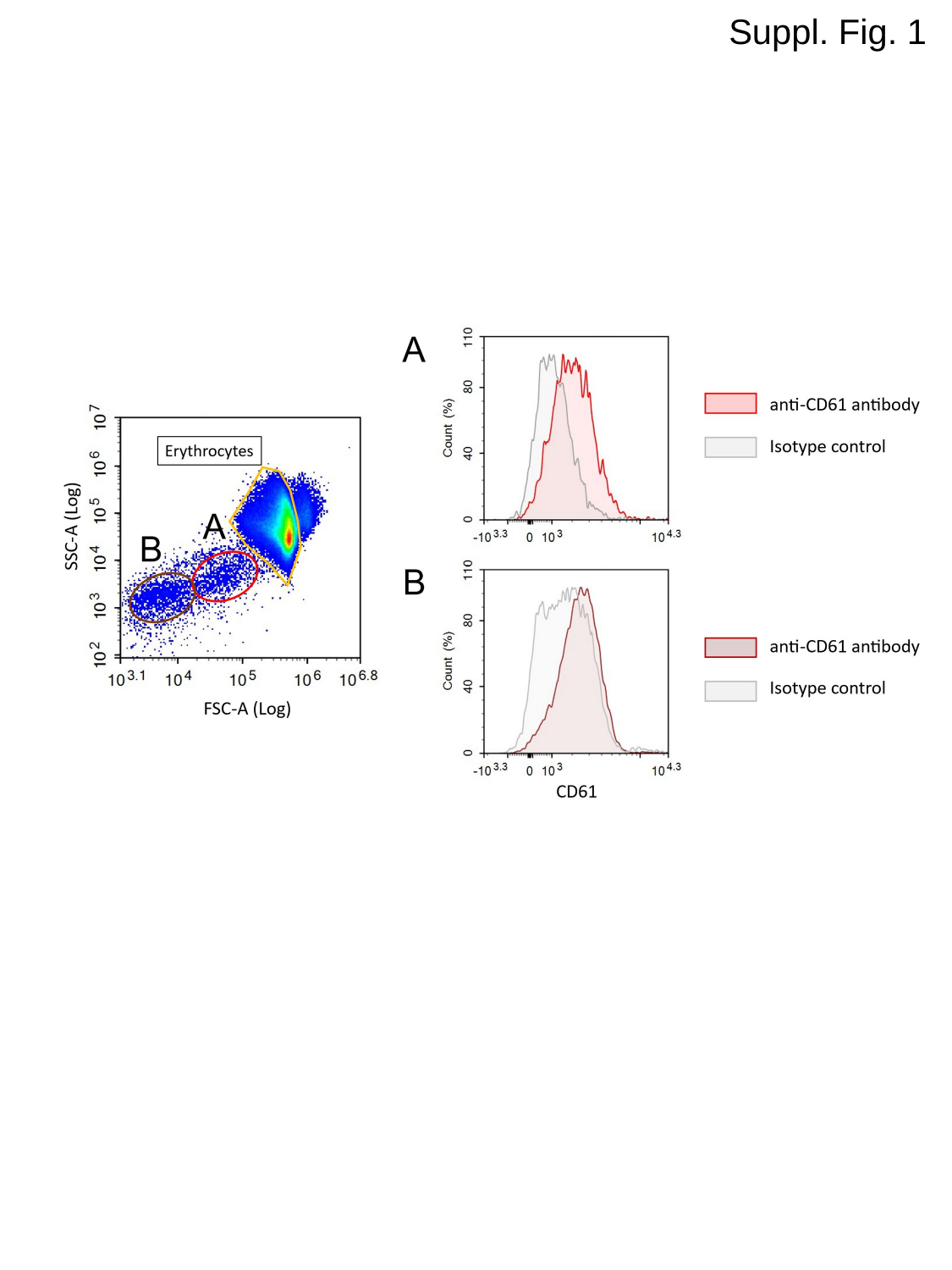

Suppl. Fig. 1

## Slide 2
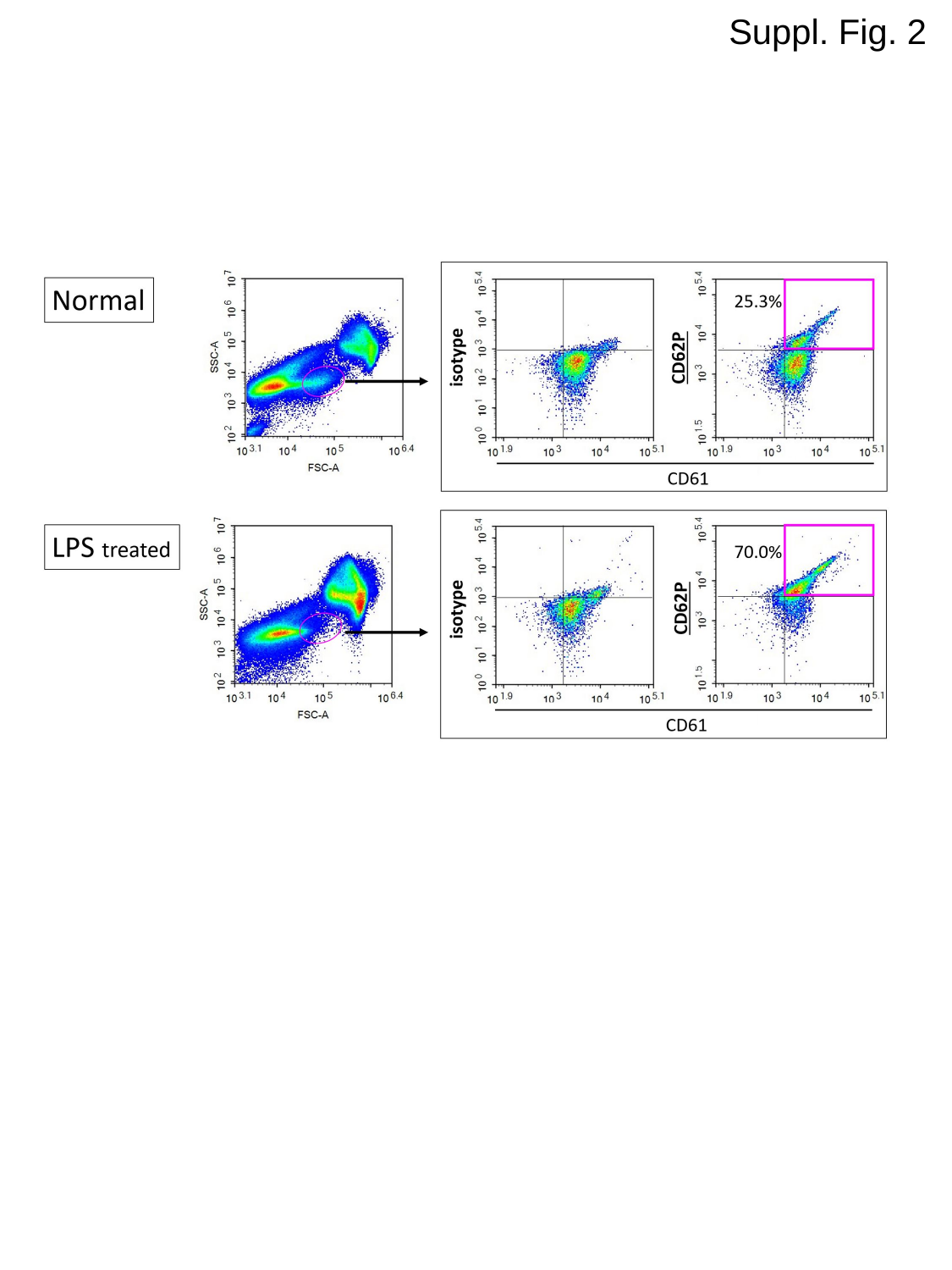

Suppl. Fig. 2

## Slide 3
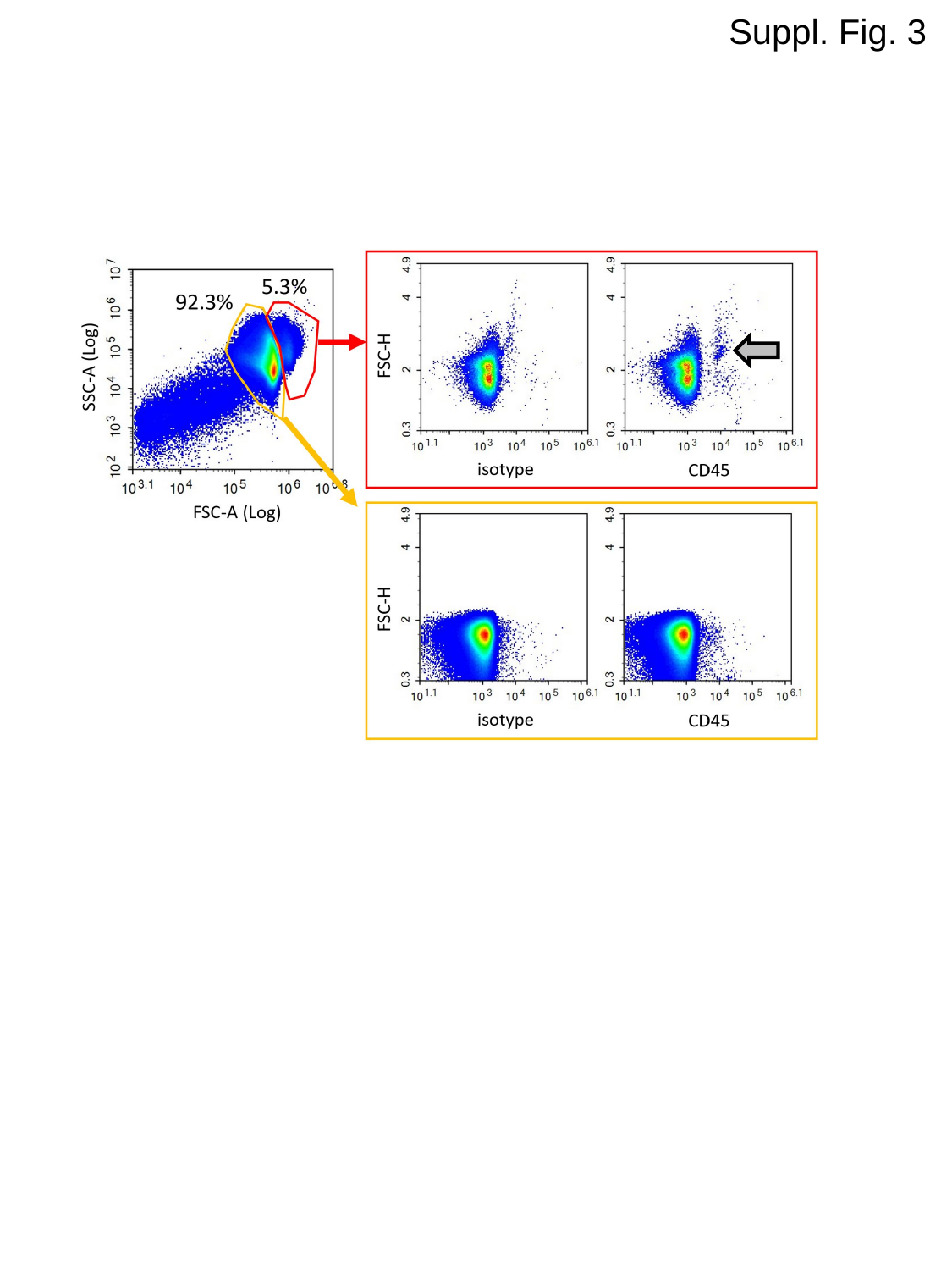

Suppl. Fig. 3

## Slide 4
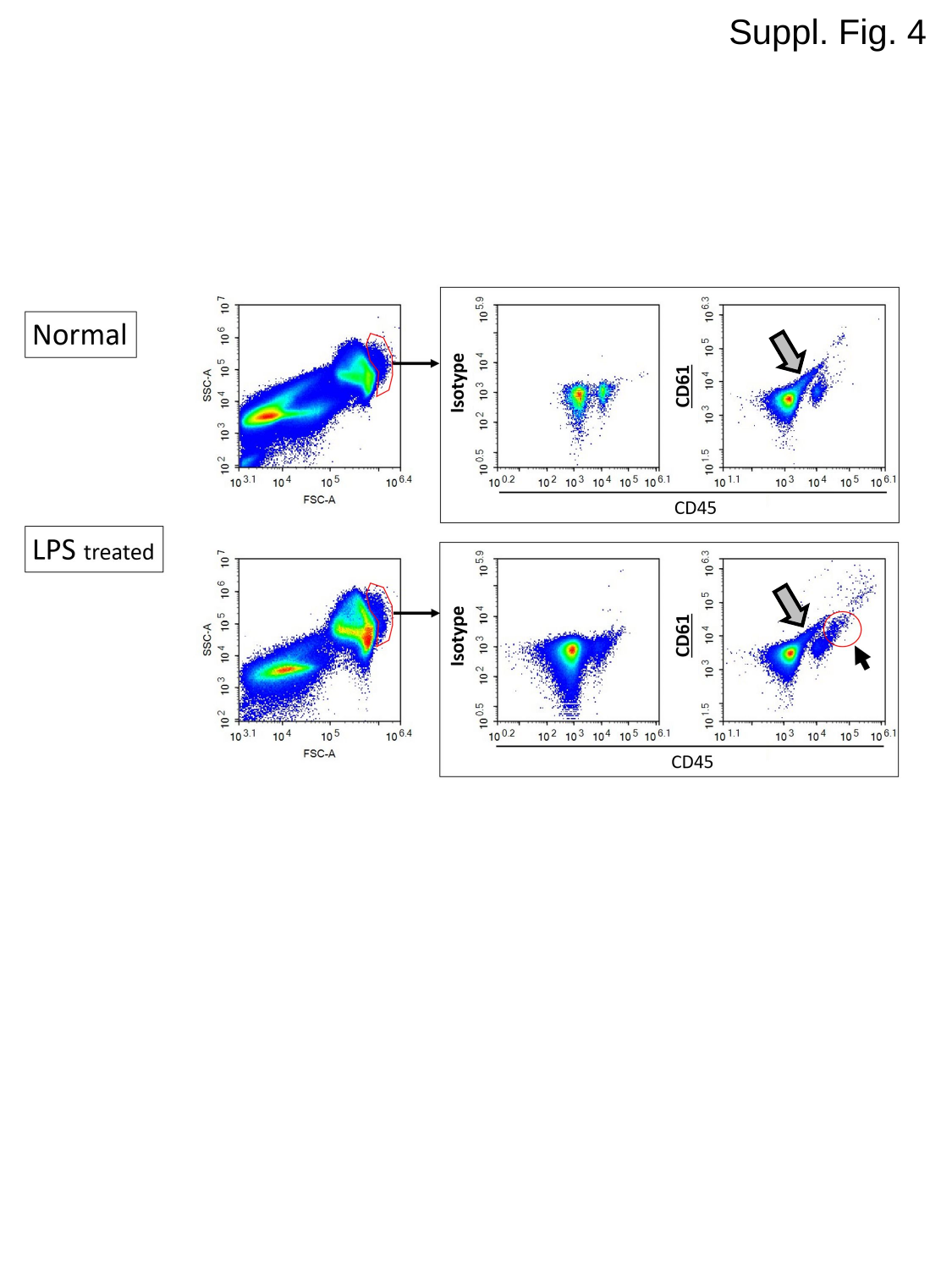

Suppl. Fig. 4

## Slide 5
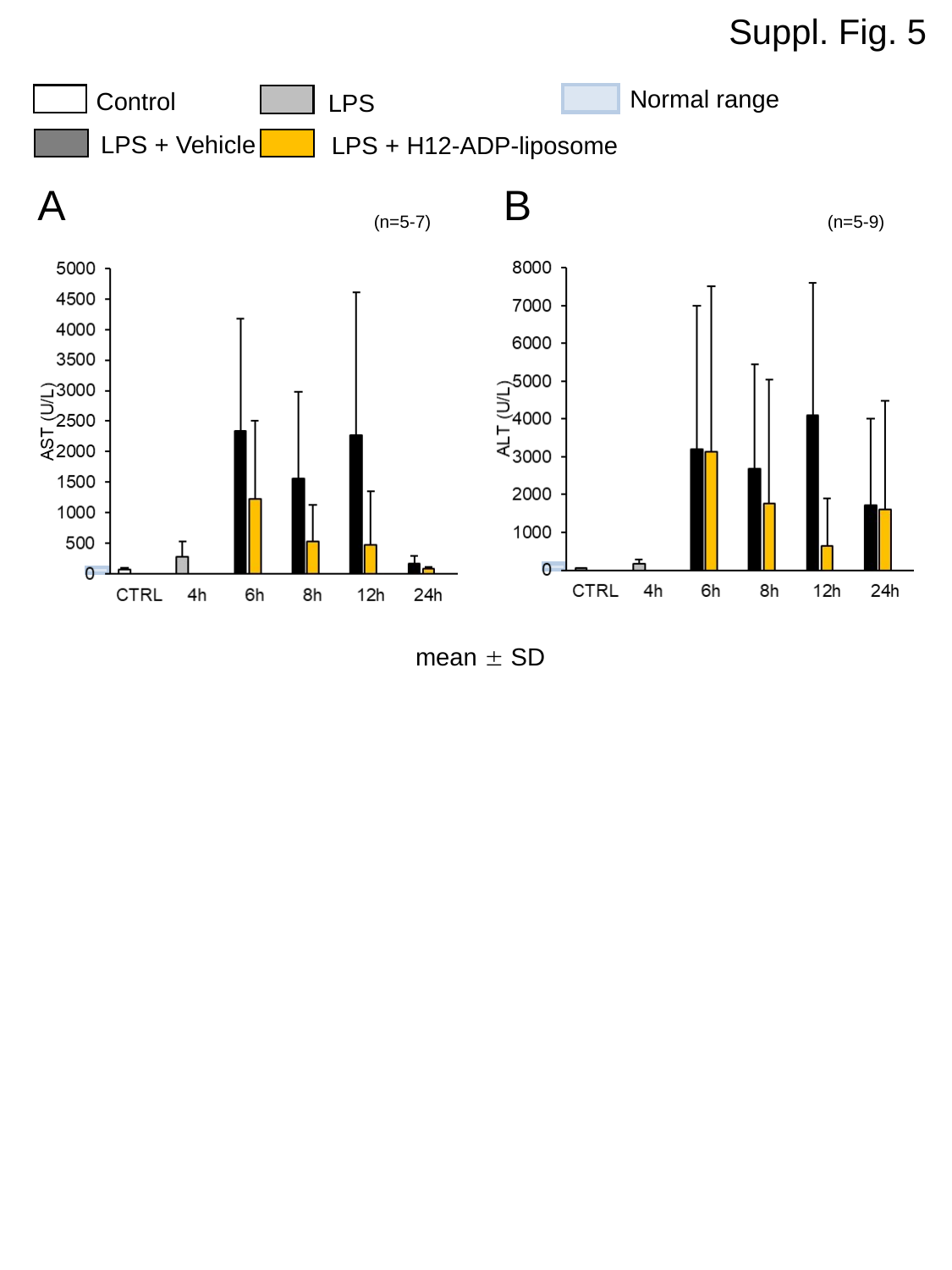

Suppl. Fig. 5
Normal range
Control
LPS
LPS + Vehicle
LPS + H12-ADP-liposome
A
B
(n=5-7)
(n=5-9)
mean ± SD
